# Long-Term Functional and Anatomical Outcome after Descemet Stripping Automated Endothelial Keratoplasty: A Prospective Single-Center Study

**DOI:** 10.1155/2018/7320816

**Published:** 2018-03-11

**Authors:** Jeroen van Rooij, Angela Engel, Lies Remeijer, Hugo van Cleijnenbreugel, René Wubbels

**Affiliations:** ^1^The Rotterdam Eye Hospital, Rotterdam, Netherlands; ^2^Rotterdam Ophthalmic Institute, Rotterdam, Netherlands; ^3^Mediclinic, Oud-Heverlee, Belgium

## Abstract

**Purpose:**

To investigate the long-term anatomical and functional outcomes of Descemet stripping automated endothelial keratoplasty (DSAEK).

**Methods:**

Prospective follow-up of 114 eyes (95 subjects) after DSAEK for endothelial dysfunction. Measurements included best spectacle-corrected visual acuity (BSCVA), straylight, endothelial cell density (ECD), and graft thickness.

**Results:**

The mean follow-up time was 5.1 ± 1.5 years. Four grafts ultimately failed (after 5 to 7 years). From baseline up to 1 year after DSAEK, mean BSCVA improved by 0.30 logMAR. This beneficial effect remained until the last follow-up (LFU). After DSAEK, straylight was reduced. ECD sharply dropped by 900 cells/mm^2^ (33%) immediately after surgery and, thereafter, steadily decreased at a rate of 11 cells/mm^2^ per month. No significant correlation was observed between graft thickness at 3 years and BSCVA.

**Conclusions:**

We observed a low graft failure rate and a normalization of graft thickness. Postoperative straylight remained elevated relative to the normal population. The sharp initial and the subsequent more gradual ECD decline are consistent with other studies. A significant and prolonged functional gain can be achieved by posterior lamellar grafting for endothelial dysfunction.

## 1. Introduction

Conditions such as Fuchs' endothelial dystrophy (FED) or pseudophakic bullous keratopathy (PBK) may lead to irreversible corneal edema and, consequently, loss of vision. Currently, FED is the most frequently registered indication for corneal transplants [1]. Full-thickness corneal surgery like penetrating keratoplasty (PKP) has some disadvantages such as an unpredictable refractive outcome and a susceptibility to trauma.

In 1956, endothelial keratoplasty was introduced for the first time [2]. Due to major innovative steps (e.g., [3–5]) and further refinement (e.g., [6, 7]), posterior lamellar techniques such as Descemet stripping automated endothelial keratoplasty (DSAEK) have become the mainstay for the surgical treatment of endothelial dysfunction and, thereby, restoration of vision [8–11]. Endothelial keratoplasty has some obvious advantages, such as small incision surgery (reducing complication rates), sutureless attachment of the donor graft to the recipient cornea (minimizing induced astigmatism), and accelerated visual recovery.

A possible side effect of the favourable results of this type of corneal transplantation as compared to penetrating grafts is that ophthalmologists and their patients may be prone to consider such an intervention as desirable at a substantially earlier stage of the condition's progression. This demands a continuous, close, and long-term surveillance of postoperative proceedings in order to allow an adequate evaluation of the benefit/risk ratio of the surgical intervention. In this study, we assessed the anatomical and ophthalmic characteristics of the donor grafts and the recipient eyes that underwent DSAEK up to 7 years after surgery.

## 2. Methods

### 2.1. Study Design and Subjects

Eligible for inclusion were patients with FED, PBK, or secondary endothelial decompensation. The subjects in this study underwent DSAEK in the Rotterdam Eye Hospital (REH). All clinical data were collected prospectively. For 56 patients, these data were retrieved from the ongoing national survey of corneal transplants of the Netherlands Organ Transplant Registry (NOTR). The ethical committee concluded that the Dutch Medical Research Involving Humans Act (WMO) did not apply to this part of the study, and therefore, official approval was not required. For 39 patients, data were collected as part of a prospective study for which approval was obtained from the Medical Ethical Committee of the Erasmus Medical Center, Rotterdam. All these 39 subjects gave written informed consent in advance. The study adhered to the tenets of the Declaration of Helsinki.

### 2.2. Surgical Procedure

Surgery was performed by 3 experienced surgeons (Hugo van Cleijnenbreugel, Lies Remeijer, and Jeroen van Rooij) between June 2007 and October 2011. Donor corneas (diameter 8.5 mm) were supplied by the Euro Cornea Bank (Beverwijk, The Netherlands) and prepared by the surgeon. A Moria microkeratome (Moria International, Antony, France) equipped with a 350 *μ*m head and a Barron Punch trephination system (Katena Products, Denville, NJ, USA) were used for the preparation of the donor lamellae. The graft was inserted either with the aid of a 10-0 prolene suture [12] or with the Busin DSAEK glide [13]. For a description of the surgical procedure in more detail, see van Cleynenbreugel and coworkers [14, 15].

### 2.3. Postoperative Medication

After surgery, the following regimens of dexamethasone (0.1%) topical eye drops were prescribed: week 1–4, 6 gtt daily; week 5–12, 4 gtt daily; week 13–52, 3 gtt daily; and thereafter, 1 gtt per day.

### 2.4. Outcomes

The best spectacle-corrected visual acuity (BSCVA) was measured using an ETDRS chart. If such data were not available, supplemental visual acuity outcomes (assessed on an angular chart projector and converted to logMAR scores) were used.

Intraocular straylight, expressed as log(s), was measured with the C-Quant instrument (Oculus GmbH, Wetzlar, Germany) by means of the compensation comparison method [16]. If the measurement was qualified as unreliable, it was rejected for further analysis [17].

Baseline endothelial cell density (ECD) from the donor cornea was provided by the Euro Cornea Bank: the trypan blue stained tissue was inspected by light microscopy, and cells were manually counted [18]. Postoperative ECD was determined by confocal microscopy (Confoscan 4; Nidek Technologies, Padova, Italy) or by specular microscopy (Topcon SP-1P, Topcon Corp., Tokyo, Japan). The best endothelial cell layer image was selected, and after outlining a region of interest, all cells within that area were marked and counted manually.

Central corneal thickness was estimated from rotating Scheimpflug images (Pentacam HR, Oculus, Wetzlar, Germany). When Scheimpflug images were missing, data were supplemented with ultrasound pachymetry (Tomey SP-100, Nürnberg, Germany).

Postoperative lamellar thickness was obtained from confocal microscopic scans. The interface between a donor lamella and a recipient cornea is designated by a layer of highly reflective particles; for establishing graft thickness, the confocal image was chosen that showed this layer most distinctly. As it is assumed that the donor tissue will have reached a stable state after a sufficient elapse of time, lamellar thickness was examined at 36 months or, if not available from that visit, at the previous or next moment of follow-up (i.e., at 24 or at 60 months).

### 2.5. Statistical Analysis

Data were analysed with SPSS (version 21, IBM Corp., Armonk, NY, USA) and are presented—on a per eye basis—at baseline and consecutive postoperative visits. Postoperative visits were scheduled at 1, 3, and 6 months and at 1, 2, 3, 5, and 7 years. Nominal data are given as the numbers and percentages; continuous data are reported as the means together with their standard deviation (SD) or 95% confidence interval (CI).

For each separate eye and for each outcome parameter, the final follow-up value was selected to obtain a maximally complete set of “last follow-up” (LFU) data. Spearman's *ρ* was calculated to inspect correlation between parameters. For the comparison of patient subgroups, an independent *t*-test or one-way ANOVA was used.

## 3. Results

In total, 114 pseudophakic eyes (95 patients) that underwent DSAEK were included in this study. Demographic (ocular-based) characteristics at baseline are listed in Table 1. Patients' age at surgery averaged 69.0 (50–86) years. Ninety-five percent (*n* = 108) of the eyes had Fuchs' endothelial dystrophy. Before surgery, the mean visual acuity was 0.48 ± 0.27 logMAR, the mean corneal thickness was 683 ± 95 *μ*m, and the mean preoperative ECD of the donor cornea was 2736 ± 153 mm^−2^ (Table 2). Straylight measurements were attempted at baseline for 60 eyes. Forty-five of these assessments were considered to be reliable and were used to calculate their mean: log(s) = 1.53 ± 0.22 (Table 2).

**Figure 1:**
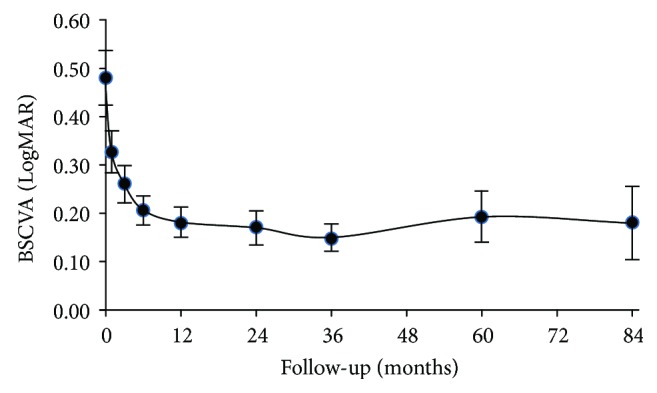
Mean best spectacle-corrected visual acuity (BSCVA) at baseline and during follow-up. Error bars represent the 95% CI.

**Figure 2:**
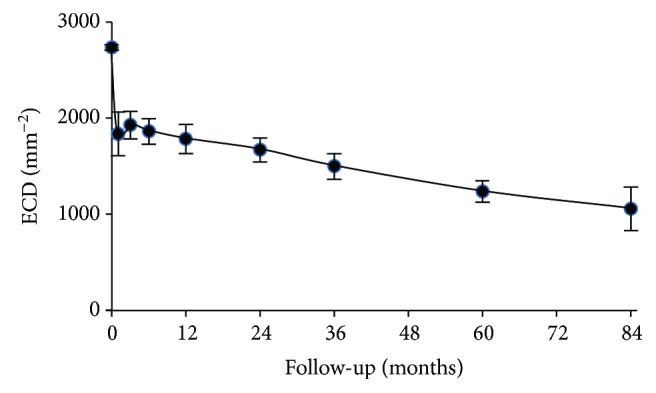
Mean endothelial cell density (ECD) at baseline and during follow-up. Error bars represent the 95% CI.

The mean follow-up time for visual acuity was 5.1 ± 1.5 years. On average, follow-up times for ECD, corneal thickness, and straylight were of similar duration. Table 2 shows the outcomes of BSCVA, ECD, pachymetry, and straylight for each visit; the former two outcomes of which are also visualized in Figures 1 and 2 (BSCVA and ECD, resp.).

Visual acuity improved substantially after DSAEK and appeared to stabilize after about 6 months postoperatively. In particular, before transplantation and during the first months of follow-up, many straylight measurements are missing or unreliable (supposedly due to the combination of poor vision and the concentration which this subjective method requires). Although one-way ANOVA analysis of the straylight outcomes (of baseline and of 1, 2, 3, 5, and 7 years postop) suggests a significant effect (*F*(5,315) = 3.06; *p* = 0.01), no clear separation of homogenous subsets was observed.

After a sharp drop of the mean ECD of about 900 cells/mm^2^ (33%) immediately after surgery, a more gradual decline is observed of 11 cells/mm^2^ per month which continues rather linearly (*R*^2^ = 0.18) during the entire follow-up. Three years after transplantation (range 1.9 to 5.3 years), the thickness of the donor lamella was 109 ± 35 *μ*m (range 38 to 220 *μ*m; *n* = 105). After slow endothelial decompensation, four grafts ultimately failed at 59, 60, 73, and 84 months. Primary graft failures did not occur in this study group. Concomitant ocular disorders potentially affecting visual acuity were observed in 24 eyes: age-related macular degeneration (AMD, *n* = 20), glaucoma (*n* = 3), and macular hole (*n* = 1). In two of these eyes (one with AMD and the other with glaucoma), graft failure occurred.

After exclusion of graft failures, the LFU analysis results were as follows: visual acuity, 0.18 ± 0.19 logMAR (*n* = 110); ECD, 1296 ± 569 mm^−2^ (*n* = 108); total corneal thickness (including the graft), 601 ± 53 *μ*m (*n* = 110); and straylight, log(s) = 1.43 ± 0.19 (*n* = 88; 108 attempted measurements). A comparison of the visual outcome of patients with and without concomitant ocular pathology showed that vision is affected indeed: 0.32 ± 0.24 logMAR (*n* = 22) versus 0.15 ± 0.16 logMAR (*n* = 88; independent *t*-test, *p* = 0.0001). The correlation (Spearman's *ρ*) between the following parameters was determined: preoperative ECD, lamellar thickness at 3 years, LFU BSCVA, LFU ECD, LFU corneal thickness, and LFU straylight. With the exceptions of *ρ* = –0.22 (*p* = 0.02) between BSCVA and corneal thickness and *ρ* = 0.44 (*p* = 0.000003) between donor pachymetry and LFU pachymetry, no significant correlation was observed.

## 4. Discussion

While most studies on corneal transplantation report excellent graft survival data, the same literature is generally less clear about functional (e.g., BSCVA) and anatomical outcomes (e.g., ECD). Our study was designed to acquire both long-term functional and anatomical outcomes of DSAEK in a large cohort.

As can be expected from the primary indication for DSAEK being the restoration of visual acuity, a significant beneficial effect on BSCVA was observed. The BSCVA values we measured at 1 year were at least comparable to those reported by other investigators [9, 10, 19, 20]. From Table 2 and Figure 1, it can be inferred that BSCVA has reached near maximum improvement after 6 months. From then on, visual acuity appears to remain stable up to 7 years of FU.

Relative to preoperative values, postoperative straylight is reduced. Similar results, both pre- and postoperatively, have been reported before for endothelial keratoplasty with FED [21]. Compared to the age-matched, normal population, however, postoperative straylight remains elevated [22]. Preoperative causes of straylight such as endothelial guttata or plaques and stromal edema are supposed to disappear postoperatively: descemetorhexis removes anatomical irregularities, and the normalization of the recipient corneal thickness (see below) can be interpreted as the resolution of stromal edema. As all patients were pseudophakic and treated with an iridium YAG laser for posterior capsule opacification when necessary [23], elevated straylight is presumed to be due to corneal haziness, possibly caused by interface opacifications or folds in the DSAEK graft.

For most eyes of this study, reliable postoperative images of the endothelium could be obtained by means of confocal microscopy. Such images permit manual ECD assessment (Table 2, Figure 2) which is generally regarded as the gold standard [24, 25]. A rapid ECD decline immediately after endothelial keratoplasty has been reported before [e.g., [26]]. ECD loss in the current study and that in another large cohort study [27] were strikingly comparable (at 1 year: 35% versus 37%, 3 years: 45% versus 44%, and 5 years: 55% versus 53%).

Thickness measurements from the confocal scan images show that after DSAEK the recipient cornea returns to normal dimensions, indicating adequate corneal endothelial pump function. We did not find any significant correlation between donor lamella thickness at 3 years and LFU BSCVA. This is in line with the conclusion of a meta-analysis [28] that graft thickness accounts for only a small part of the variance in visual outcome. In contrast to this meta-analysis, however, which only involved prospective/retrospective cohort studies and case series, a properly designed randomized study comparing conventional DSAEK (mean graft thickness 209 *μ*m) to ultrathin DSAEK (mean graft thickness 101 *μ*m) reports a clear effect of thickness on BSCVA [20].

Close inspection of Figure 2 by Dickman and coworkers [20] on the other hand learns that the average visual improvement is −0.11 logMAR for DSAEK and −0.14 logMAR for ultrathin DSAEK. Therefore, we believe one should be careful with respect to inferring a relevant clinical benefit from a statistically significant effect. Future adequately powered comparative trials may provide a more conclusive answer with respect to the import of both the statistical and the clinical significance of graft thickness on visual outcome.

## 5. Conclusion

The early postoperative decline in graft endothelial cell density we observed is reported by other studies as well. The (subjective) straylight measurements appear to indicate no more than just a weak improvement after DSAEK. With respect to visual function, however, we conclude that our results present additional evidence for the substantial gain that can be achieved by posterior lamellar grafting for endothelial dysfunction, in particular, in the eyes with Fuchs' endothelial dystrophy. The average visual gain was 0.3 logMAR after 1 year, a clinically beneficial effect which appears to last for at least up to 7 years. Graft failure was low (3.5%).

## Figures and Tables

**Table 1:** Demographic data—on a per eye basis—at baseline; the number of patients was 95.

Gender	
Male	56 (49%)
Female	58 (51%)
Eye	
Right	62 (54%)
Left	52 (46%)
Age at surgery	69.0 ± 7.9
Indication for DSAEK	
FED	108 (95%)
PBK	4 (4%)
Decompensation/primary graft failure	2 (2%)

DSAEK: Descemet stripping automated endothelial keratoplasty; FED: Fuchs' endothelial dystrophy; PBK: pseudophakic bullous keratopathy.

**Table 2:** Ophthalmic and anatomical outcomes (means ± SD) and the number of eyes (*n*) that were assessed at baseline and during follow-up.

	Baseline	1 month	3 months	6 months	1 year	2 years	3 years	5 years	7 years
BSCVA (logMAR)	0.48 ± 0.27 *n* = 90	0.33 ± 0.18 *n* = 69	0.26 ± 0.17 *n* = 77	0.21 ± 0.13 *n* = 80	0.18 ± 0.13 *n* = 70	0.17 ± 0.16 *n* = 83	0.15 ± 0.14 *n* = 93	0.19 ± 0.26 *n* = 94	0.18 ± 0.20 *n* = 27
Straylight (log(s))	1.53 ± 0.22 *n* = 45 (60)^a^	1.40 ± 0.24 *n* = 19 (26)	1.44 ± 0.24 *n* = 27 (31)	1.40 ± 0.17 *n* = 27 (34)	1.41 ± 0.15 *n* = 44 (51)	1.38 ± 0.25 *n* = 64 (73)	1.43 ± 0.17 *n* = 77 (91)	1.44 ± 0.21 *n* = 69 (82)	1.45 ± 0.19 *n* = 22 (24)
Graft ECD (cells/mm^2^)	2736 ± 153 *n* = 108	1837 ± 567 *n* = 24	1929 ± 583 *n* = 64	1864 ± 521 *n* = 61	1783 ± 609 *n* = 62	1672 ± 552 *n* = 79	1501 ± 607 *n* = 84	1237 ± 524 *n* = 86	1058 ± 587 *n* = 26
Corneal thickness (*μ*m)	683 ± 95 *n* = 97	595 ± 50 *n* = 69	592 ± 50 *n* = 94	592 ± 47 *n* = 89	589 ± 46 *n* = 82	588 ± 46 *n* = 76	587 ± 48 *n* = 92	610 ± 87 *n* = 94	617 ± 58 *n* = 35

SD: standard deviation; BSCVA: best spectacle corrected visual acuity; ECD: endothelial cell density. ^a^In parentheses, the number of eyes for which straylight measurements were attempted.
